# Chemically Stable Styrenic Electrospun Membranes with Tailorable Surface Chemistry

**DOI:** 10.3390/membranes13110870

**Published:** 2023-11-02

**Authors:** Maura Sepesy, Tuli Banik, Joelle Scott, Luke A. F. Venturina, Alec Johnson, Bernadette L. Schneider, Megan M. Sibley, Christine E. Duval

**Affiliations:** Department of Chemical and Biomolecular Engineering, Case Western Reserve University, Cleveland, OH 44106, USA

**Keywords:** microfiltration, adsorptive chromatography, non-woven membranes, electrospinning, chloromethyl styrene, polyvinyl benzyl chloride

## Abstract

Membranes with tailorable surface chemistry have applications in a wide range of industries. Synthesizing membranes from poly(chloromethyl styrene) directly incorporates an alkyl halide surface-bound initiator which can be used to install functional groups via S_N_2 chemistry or graft polymerization techniques. In this work, poly(chloromethyl styrene) membranes were synthesized through electrospinning. After fabrication, membranes were crosslinked with a diamine, and the chemical resistance of the membranes was evaluated by exposure to 10 M nitric acid, ethanol, or tetrahydrofuran for 24 h. The resulting membranes had diameters on the order of 2–5 microns, porosities of >80%, and permeance on the order of 10,000 L/m^2^/h/bar. Crosslinking the membranes generally increased the chemical stability. The degree of crosslinking was approximated using elemental analysis for nitrogen and ranged from 0.5 to 0.9 N%. The poly(chloromethyl styrene) membrane with the highest degree of crosslinking did not dissolve in THF after 24 h and retained its high permeance after solvent exposure. The presented chemically resistant membranes can serve as a platform technology due to their versatile surface chemistry and can be used in membrane manufacturing techniques that require the membrane to be contacted with organic solvents or monomers. They can also serve as a platform for separations that are performed in strong acids.

## 1. Introduction

Membrane-based separation processes are applied to a wide range of separation challenges including saltwater desalination, organic solvent separations, pharmaceutical processing, wastewater remediation, and more. Over the past 60 years, implementing membrane-based solutions has benefited these industries in different ways. For example, membrane-based desalination is 10× more energy efficient than classical distillation [1]. CO_2_ capture with membranes has a smaller physical footprint than traditional scrubbing units [2]. Additionally, membrane-based chromatography processes have shorter purification times and smaller bed volumes than their resin equivalents [3]. For these reasons, the membrane community seeks to develop membranes for emerging applications such as organic solvent separations, pervaporation to remove oil from brackish fracking water, and contaminant removal or resource recovery. Chemical stability is necessary for membranes to withstand harsh chemical environments in applications such as organic solvent filtration [4], pervaporation [5], and radiochemical separations [6]. Further, in many membrane functionalization strategies, polymeric membranes are exposed to organic solvents or organic ligands that can impact membrane morphology. Without requisite chemical stability, membranes can deform or dissolve.

One strategy to enhance the chemical stability is to introduce chemical or physical crosslinking [7,8]. Lin et al. synthesized cross-linked anion exchange membranes by introducing the crosslinking agent (tetraethylenepentamine) to the monomer solution (chloromethyl styrene and divinylbenzene) prior to casting the membrane [7]. The crosslinked membranes demonstrated improved alkaline stability and improved ionic conductivity compared to the non-crosslinked counterparts [7]. Alternatively, membranes can be crosslinked after fabrication to enable control over the membrane structure. Ong et al. prepared branched polyethyleneimine or 1,4-butanediol diglycidyl ether crosslinked polycarbonate track-etched microfiltration membranes via a two-step process after membrane fabrication [9]. These membranes were exposed to acetone, toluene, dimethyl sulfoxide, ethyl acetate, and isopropyl alcohol for seven days at room temperature [9]. Chemical stability tests showed that while most of the pristine membranes dissolved in the solvents, none of the 10% crosslinked membranes dissolved [9]. 

Microfiltration membranes are used for size-based separation processes and as scaffolds for adsorptive separations, and they can be synthesized through phase inversion processes or electrospinning [10]. A persistent challenge with membrane adsorbers synthesized using either method is the limited maximum capacity when compared to commercial resins. Compared to phase inversion membranes, electrospun membranes have higher porosities which enable high permeance and may have a higher surface area depending on the fiber diameter. Despite the lower maximum capacities, membrane adsorbers have made recent advances in applications that prioritize smaller bed volumes, have dilute feeds, and require high volumetric flowrates, including radioanalytical chemistry [11,12,13], water treatment [14], and biologics purification [15]. A basic electrospinning apparatus consists of a high-voltage power supply, a syringe with a needle, a syringe pump, and a fiber collector [16,17,18,19,20,21]. During an electrospinning experiment, a polymer solution is pumped through the syringe, charged by the power supply, and ultimately attracted to the grounded collector when the electrostatic attraction exceeds the surface tension of the Taylor cone. This process creates a continuous stream of polymer that spins itself into a fibrous mat [16]. There are many electrospinning variables that can affect fiber formation and morphology, including the applied voltage, distance between the needle and the collector, polymer concentration of the dope [17], spinning time [18], flow rate, and humidity [16,19]. Also, the conductivity of the polymer dope impacts the spinning process. For example, adding 0.5–1 wt% of sodium chloride, lithium chloride, or sodium dodecyl sulfate increased the conductivity of 8–15 wt% polystyrene/DMF solutions, 4–10 wt% polyacrylonitrile/DMF solutions, and 3–7 wt% poly(ethylene oxide)/water solutions [19,21,22] and ultimately improved the spinnability of the polymer dope solutions. Without the addition of salt, the tested polymer dope solutions were electrosprayed from the needle tip, creating particles rather than fibers. While lab-scale efforts are common, scaling up electrospinning processes for roll-to-roll manufacturing is more challenging than a standard phase-inversion membrane manufacturing process due to the high voltage power sources, volatilization of solvents, and more complicated process equipment.

After fiber formation, it is possible to further tailor the membrane surface to increase fouling resistance, improve selectivity, or improve bio-compatibility. Three common techniques for adding functional polymer coatings to membranes are (1) gamma-induced free-radical polymerization, (2) reversible-addition fragmentation chain transfer (RAFT), and (3) surface-initiated atom transfer radical polymerization (SI-ATRP). The irradiation of polyethylene with gamma rays initiates an uncontrolled free-radical polymerization by splitting the polymer backbone, thereby generating free radicals. Then, grafted brushes are grown as monomers propagate from the main chain [22]. The irradiation of polyethylene affords little control over the polydispersity of the grafted brushes [23], with a typical polydispersity index (PDI) of around 10 [24]. Furthermore, two active chains can combine to terminate polymerization, which results in a crosslinked polymer [22]. Other surface-initiated polymerization techniques, such as free-radical polymerization or UV-induced polymerization, have poor control over the propagation step in the reaction, which determines the film thickness and uniformity. Some of these shortcomings are addressed by using controlled polymerization like RAFT or SI-ATRP. RAFT uses thiocarbonylthio mediators to introduce a rapid, reversible reaction between “dormant” and “active” chains [25]. Chosen carefully, the mediator can provide equal probability for growth on each grafted chain. For most polymers, the PDI ranges from 1.05 to 1.4 [24]. RAFT also boasts scalability, readily available reagents, and compatibility with a wide range of monomers and polymer systems [23]. It has been used to functionalize membranes made from polyethylene, cellulose acetate, polystyrene, and poly(chloromethyl styrene) [25]. In SI-ATRP, alkyl halide end groups are used as ATRP initiators for grafting from the membrane surface [26]. Like RAFT, SI-ATRP allows for controlled film thickness, composition, and macromolecular architecture due to its slow polymerization [27]. Surface-bound initiators can be installed via polyelectrolyte complexation [27] or covalent bonding to the membrane surface [28,29,30]. Alternatively, membranes synthesized from polymers containing ATRP initiators can provide a direct route for grafting from the membrane. For example, fibers containing bromine-terminated polymers, such as polystyrene after reaction with α-methylbenzyl bromide, can be used as solid-phase initiators to graft polymer chains from the surface [28,31,32,33].

Beyond grafting from the membrane surface, other techniques can be used to directly install a single functional group or functional polymer. Click chemistry is another means of attaching polymer brushes to electrospun fibers. Kolb et al. defined this term to classify reactions meeting a set of requirements including a high yield, stereospecificity, readily available reagents, a modular synthesis, and an easily scalable separation [34]. Click chemistry is attractive for membrane-surface modification since (1) it enables chemists to add covalent bonds in a predictable, stepwise fashion, and (2) typical rate constants provide good scalability toward the eventual mass production of membranes [35]. In particular, the Huisgen cycloaddition between alkynes and azido groups is useful for functionalizing membranes with alkyne-terminated monomers. Fu et al. coupled alkyne-terminated poly(N-isopropylacrylamide) (poly(NIPAM)) to poly(glycidyl methacrylate) (poly(GMA)) and poly(chloromethyl styrene) (PCMS) [36] membranes using this technique. First, poly(GMA)-*block*-PCMS was electrospun to create fiber mats. After spinning, membranes were treated with sodium azide, populating the surface with azido groups which allowed for cycloaddition with the alkyne group on poly(NIPAM) [36]. A distinct advantage of the azide-alkyne reaction lies in its exceptional selectivity, and it is regarded as one of the most useful click chemistry reactions [34]. Unfortunately, the utility of click chemistry is limited to polymer systems in which the requisite functional groups can be installed.

A common theme for the controlled polymerization techniques (RAFT, SI-ATRP) and click chemistry is the need to install an initiator on the surface. In particular, the chloromethyl moiety of chloromethyl styrene can be leveraged for a wide range of functionalization reactions, including graft polymerizations and S_N_2 reactions. Herein, we report the synthesis of electrospun membranes formed from blends of poly(styrene) and poly(chloromethyl styrene) as well as pure poly(chloromethyl styrene). These membranes have tailorable surface chemistries amenable to functionalization via RAFT, SI-ATRP, or click chemistry through the chloromethyl moiety on the PCMS polymer chains. Further, we evaluate the chemical stability of the membranes and later improve the chemical stability through a post-fabrication crosslinking reaction using a diamine. Importantly, this work provides strategies for increasing the chemical stability of electrospun membranes while maintaining sufficient reactive sites for future functionalization. This strategy may enable their use in a wide range of applications that require exposure to organic solvents in membrane manufacturing or the final applications.

## 2. Materials and Methods

### 2.1. List of Materials and Reagents

To create polymer blends for electrospinning, polystyrene (PS, M_w_ 350,000) and poly(chloromethyl styrene) (PCMS, M_w_ 100,000) were purchased from Sigma-Aldrich (St. Louis, MO, USA). Pure PCMS membranes were synthesized using PCMS (M_w_ 500,000) purchased from Scientific Polymer Products (Ontario, NY, USA). The molecular weights were reported by the manufacturers. Lithium chloride (99+%, ACS reagent grade, Acros Organics, Beel, Belgium) and tetrahydrofuran (certified, Fisher, Waltham, MA, USA) were used in the spinning solution. N,N-dimethyl ethylene diamine (DMEDA, Alfa Aesar, Ward Hill, MA, USA), potassium carbonate (Fisher), and potassium iodide (Fisher, Waltham, MA, USA) were used in the crosslinking reactions. Porosity measurements were performed using 2-propanol (certified ACS Plus, Fisher, Waltham, MA, USA). Chemical resistance tests were performed using the following chemicals: ethanol (reagent grade, Honeywell, Charlotte, NC, USA), nitric acid (HNO_3_, certified ACS Plus, 15.8 M, Fisher), and tetrahydrofuran (THF, certified, Fisher, Waltham, MA, USA).

### 2.2. Preparing the Polymer Dope for Electrospinning

Two types of polymer dope solutions were prepared. The first type contained a blend of two polymers, PCMS, and PS, and the second type contained pure PCMS. At the beginning of this investigation, the research team only had access to the lower-molecular-weight PCMS. To increase the average molecular weight of the electrospun fibers, this lower-molecular-weight PCMS (M_w_ = 100,000) was blended with a higher-molecular-weight PS (M_w_ = 350,000). Later, the higher-molecular-weight PCMS (M_w_ = 500,000) became available and was used in its pure form.

To prepare the polymer dope for the PCMS-PS blended membranes, commercially available PCMS and PS chip were dissolved in the same solvent, THF. PCMS and PS were weighed individually using an analytical balance (Explorer^TM^ Analytical, OHAUS, Parsippany, NJ, USA) to achieve the desired mass ratio of PS:PCMS of 80:20. Then, a total of 3 g of polymer chip was added to 16.8 mL of THF and 0.1 g LiCl in an amber jar with a threaded cap. The final polymer dope solution contained 15 wt% polymer and 0.5 wt% LiCl. The jars were capped and set on a shaker table (LabLine 3527 Orbital Incubator Shaker Table, Eroor South, India) at 75–100 RPM overnight for the polymer to dissolve.

To prepare the polymer dope for the pure PCMS membranes, commercially available PCMS was dissolved in THF. Specifically, 3 g PCMS and 0.1 g of LiCl were added to an amber jar containing 16.8 mL of THF. The final polymer dope solution contained 15 wt% polymer and 0.5 wt% LiCl. The capped jars were set on the shaker table and mixed overnight at 75–100 RPM.

### 2.3. Electrospinning Apparatus

A home-built, humidity-controlled electrospinning system was constructed for this work. The humidity-controlled static box was situated inside a chemical fume hood and placed on risers (8 × 8 × 4.5 cm acrylic blocks). The risers assisted with airflow within the fume hood and enabled the cabinet doors to clear the fume hood ledge. Diagrams of the electrospinning system, descriptions of its construction, and a list of complete parts are summarized in the Supporting Information.

### 2.4. Electrospinning Membranes

Prior to electrospinning, static was discharged from the system by wiping the inside of the static box with a dryer sheet (any unscented, e.g., all®, free clear, Henkel Corp., Rocky Hill, CT) and discharging a Zerostat 3 Anti-Static Gun. This process dissipates any residual static charge from previous electrospinning experiments and minimizes arcing during operation. Next, the static box was humidified to the desired set point (40–50% relative humidity). Meanwhile, 17 mL of the polymer dope was drawn into a 30 mL glass syringe (VWR, Radnor, PA, USA). A blunt-tipped, 22-gauge needle was installed on the syringe. Then, the syringe was mounted in the syringe pump, and the needle was threaded through the acrylic divider (Diagram in Supporting Information, Figure S1). In the spinning compartment (right-hand side), the system was grounded by clamping a black alligator clip to the aluminum foil-coated collector plate with Fisherbrand™ P4 filter paper (Fisher, Waltham, MA, USA) attached. The black alligator clip was grounded to the static box. Finally, the needle was connected to the power supply using a red alligator clip. Both alligator clips were 304 stainless steel; 30 A clips (McMaster-Carr, Elmhurst, IN, USA) and wires were attached using AMP Female Disconnects for 10–12 gauge wires. The needle-to-plate distance could be adjusted by varying the height of the lab jack (4–14 cm), which was measured using a ruler. Finally, the doors to the static box were closed.

Once the desired relative humidity of 50% was achieved within the static box, the syringe pump was turned on and dispensed the polymer solution at a constant flowrate of 7 mL/h. Once the first droplet of polymer solution appeared at the needle tip, the high-voltage power source was turned to 20 kV. The spinning process proceeded for 10 min. To terminate spinning, the power supply was turned off followed by the syringe pump. Finally, the doors to the static box were opened, and the system was vented to the fume hood for 5 min. After electrospinning, the filter paper-backed membranes were removed from the aluminum foil and collector plate and placed in the fume hood to air dry. The filter paper served as a support membrane and remained attached to the fibers for all subsequent experiments.

### 2.5. Crosslinking Membranes to Increase Chemical Stability

**Scheme 1:**
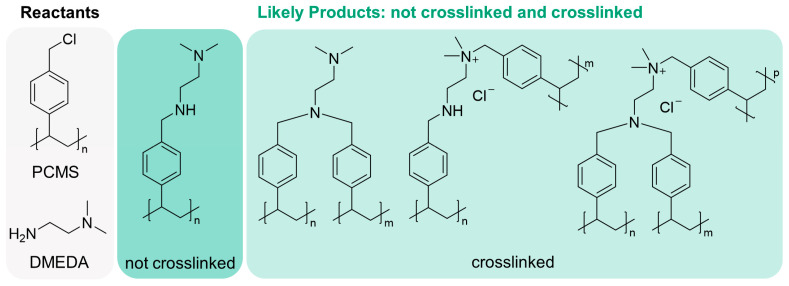
Potential crosslinking reactions which create covalent bonds between polymer chains. Here, N,N-Dimethylethylene diamine (DMEDA) was the crosslinking agent, potassium iodide was the catalyst, and potassium carbonate was the base.

Chemical crosslinking was employed as a strategy to increase chemical stability by increasing the molecular weight of the polymer chains in the electrospun membranes. Chemical crosslinking was performed on electrospun 80:20 (wt/wt) PS:PCMS and pure PCMS membranes. The crosslinking reaction time was fixed at two days, and the independent variable was the concentration of the DMEDA crosslinker, which was 40 mM, 200 mM, or 400 mM. The same mass of membrane, regardless of the PCMS content, was used for the crosslinking reactions. Thus, the molar ratio of chloromethyl styrene monomer units (CMS) to DMEDA was calculated for both membrane designs. For the pure PCMS membranes, the CMS:DMEDA molar ratios tested were 1:2, 1:9, and 1:18. For PS-PCMS blend membranes, the CMS:DMEDA molar ratios tested were 1:10, 1:48, and 1:96. The likely crosslinking products are shown in Scheme 1 below.

To perform a crosslinking reaction, the PS-PCMS blend or PCMS membrane coupon was cut using a 44 mm die punch from a MAYHEW PRO metric punch set (Turners Falls, MA, USA). Each membrane coupon used in the crosslinking reactions weighed ~0.2 g, and ~0.08 g of the membrane coupon corresponded to the mass of electrospun fibers. Meanwhile, the DMEDA was weighed in a jar with a threaded cap. Then, 0.180 g of potassium carbonate and 0.110 g of potassium iodide were added to the DMEDA-containing jar. Next, 25 mL of DI water and the membrane coupon were added to the jar. The membrane was placed in the jar with the fibers facing down. The hydrophilic filter paper backing absorbed water and aided in keeping the fibers submerged and wetted by the reaction mixture during crosslinking. Finally, the lid was placed on the jar, and the closed system was mixed on a shaker table at 150 RPM for two days. After the reaction, the crosslinked membrane was removed from the reaction mixture and placed in a clean jar containing 30 mL of DI water for 30 min to remove any unreacted or physisorbed DMEDA. Then, membranes were dried at room temperature in the fume hood for 24 h.

### 2.6. Membrane Porosity Measurements

The porosity of the membrane is the ratio of the interstitial space to the total volume of the membrane, represented as ε. Membrane porosity was measured before and after crosslinking as well as after exposure to solvents. Porosity measurements were made gravimetrically by contacting the membrane with a non-swelling solvent, isopropyl alcohol (IPA). Pristine membrane coupons (0.028 ± 0.011 g) were cut from a sheet of an electrospun membrane. The aluminum foil backing was removed prior to weighing the dry membrane. As noted previously, the filter paper backing remained for all measurements. Then, membrane coupons were submerged in 20 mL isopropyl alcohol in scintillation vials overnight on a shaker table at 75–100 RPM. After 24 h, the membranes were removed from the IPA-filled jars, immediately blotted with a Kim wipe to remove excess solvent, and weighed. The porosity is calculated according to Equation (1).

(1) $$\varepsilon =\frac{(m_{w}-m_{d})/\rho_{i}}{(m_{w}-m_{d})/\rho_{i}+m_{d}/\rho_{p}}$$

where *m_w_* and *m_d_* are the mass (g) of the wet and dry membranes, respectively, and *ρ_i_* and *ρ_p_* are the densities of IPA and the polymer (g/cm^3^). The density of PCMS was 1.088 g/cm^3^, as reported by the manufacturer. The density of the PS was 1.05 g/cm^3^, as reported by the manufacturer. The density of the PS-PCMS blend was calculated using the weighted average of the densities. Three membranes were tested, and the error associated with the measurement is represented as the standard deviation. All data that met normality requirements were analyzed using the Tukey method for ANOVA tests to determine statistical significance for any of the samples at α of 0.05. For all other non-parametric data, a Mann–Whitney test with a confidence interval of 95% was used.

### 2.7. Scanning Electron Microscopy and Fiber Diameter Measurements

The morphology of the membranes was observed using scanning electron microscopy (SEM). Samples were dried, cut, and mounted on a SEM pin stub (standard SEM pin stub mount, 12.7 mm diameter, 8 mm pin height) with carbon tape (5 mm width, Ted Pella, Redding, CA, USA). Prior to imaging, samples were sputter-coated with palladium using a Denton Vacuum DESK IV cold sputter coater (Denton Vacuum LLC, Moorestown, NJ, USA) for 30 s at 0.19 nm/s. All images were collected with a ThermoFisher Apreo 2S SEM (Waltham, MA, USA) at a working distance of 6.8–10.7 mm, a current of 6.3–50 pA, an accelerating electron voltage of 2.00 kV, and a 0° tilt. Magnification of samples ranged from 3500 to 1000×.

SEM images were imported into ImageJ 148 software (National Institutes of Health, Bethesda, MD, USA). Images were calibrated by converting pixels to microns using the scale bar on the original image. Then, the ImageJ measurement tool was used to analyze the fiber diameter. All data were non-parametric data, and a Mann–Whitney test with a confidence interval of 95% was used.

### 2.8. Solvent Exposure to Assess Chemical Stability of Membranes

Chemical stability testing provides insight into its robustness under different manufacturing conditions (solvent exposure and crosslinking). The chemical stability of the membranes was evaluated by exposing them to THF, ethanol, and 10 M HNO_3_ for 24 h. At the beginning of the experiment, membranes were cut into five pieces. Dried membranes were weighed (0.028 ± 0.011 g) and placed in a 20 mL scintillation vial containing 5 mL of a solvent (THF, 10 M HNO_3_, or ethanol). The membrane-containing jars were capped and left on the lab bench for 24 h. After 24 h, membranes were removed from the solutions, placed in a glass Petri dish, then dried in the vacuum oven under 508 mmHg vacuum until constant mass. The final mass was recorded. After exposure to the solvents, membranes were characterized to assess changes in morphology using SEM and porosity experiments according to the procedures described above.

### 2.9. Pure Water Flux Measurements

Prior to pure water flux experiments, membranes were pressed using a pneumatic laboratory press (Carver Model C 12 Ton Manual Laboratory Press 3851). Membranes were placed between two aluminum foil-coated plates (15 × 15 × 2.5 cm). The pressure was increased to 1 metric ton for 10 min.

Pure water flux experiments were performed for the membrane before crosslinking, after crosslinking, and after chemical stability testing. Membranes with a 44 mm diameter were placed in a 50 mL dead-end filtration cell (Millipore Sigma, Burlington, MA, USA). The DI water was pressurized in a 10 L water tank using a compressed air cylinder (UN1002, Airgas, Houston, TX, USA) to obtain pressures between 69 and 138 kPa (10–20 psi). The permeate was collected for 30 s and weighed using a digital balance (OHAUS Ranger 7000, OHAUS, Parsippany, NJ, USA). Using Darcy’s law as applied to pore flow membranes, the water flux and transmembrane pressure were used to calculate the membrane permeance according to Equation (2).

*J* = *A* × Δ*P* (2)

where *A* is the permeance (L/m^2^/h/bar or LMH/bar), *J* is the flux (LMH), and Δ*P* is the transmembrane pressure (bar). The permeance, *A*, may also be referred to as the permeability coefficient. When derived using the Hagen–Poiseuille model of fluid flow in a cylindrical pore, permeance is described through physical factors of the membrane and properties of the fluid as shown below in Equation (3).

(3) $$A=\frac{\varepsilon \;d_{pore}^{2}}{32\;\eta \;\tau \;l_{pore}}$$

where *ε* is the porosity, *d_pore_* is the diameter of the pore space, *η* is the dynamic viscosity of the permeate, *τ* is the tortuosity of the membrane, and *l_pore_* is the thickness of the membrane. Important for this work, the porosity is directly proportional to the membrane permeance [37]. All data that met normality requirements were analyzed using the Tukey method for ANOVA tests to determine statistical significance for any of the samples at an α of 0.05. For all other non-parametric data, a Mann–Whitney test with a confidence interval of 95% was used.

### 2.10. Elemental Analysis

Elemental analysis was performed on pristine membranes before crosslinking and membranes crosslinked with varying ratios of DMEDA to obtain the nitrogen content, represented as weight percent of N. Three membranes were analyzed to assess the reproducibility across membrane batches. The N content is a proxy for the degree of crosslinking as the DMEDA is the only species that contains N. Elemental analysis was performed by Midwest Microlabs (Indianapolis, IN) using 5 mg from each membrane sample. The analysis was performed through combustion at 990 °C using the elemental analyzer. The limit of detection for the technique, as reported by the service provider, was 0.15%N, and the error was 0.3%.

## 3. Results

### 3.1. PS-PCMS Blend Membranes

**Figure 1:**
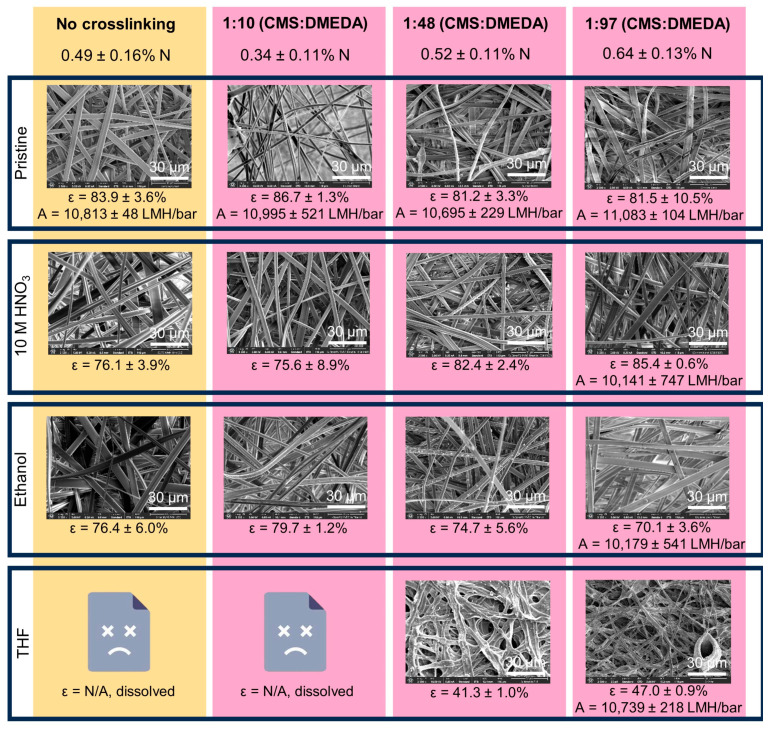
Data summary for PS-PCMS blend membranes with representative SEM images of electrospun membranes with varying degrees of crosslinking (columns) exposed to different solvents (rows). Nitrogen content (N) and porosity (ε) were measured for all membranes. Permeance (A) was measured for all pristine membranes and 1:97 CMS:DMEDA membranes after each solvent exposure. Increasing the degree of crosslinking was qualitatively seen to increase the chemical stability of the fibers.

SEM images, porosity data, and membrane permeance are summarized in Figure 1 for all synthesized PS-PCMS blend membranes.

The pristine, uncrosslinked PS-PCMS blend membrane is shown in the top left corner of Figure 1. The resulting membranes contained defect-free (e.g., bead-free) fibers with an average diameter of 4.0 ± 2.6 μm. The membranes had a porosity of 83.9 ± 3.6% and permeance (A) of 10,810 ± 48 LMH/bar. The permeance was high compared to other hydrophobic, microfiltration membranes like polyvinylidene fluoride (PVDF) where A = 5193 ± 7 LMH/bar [6]. The uncrosslinked, pristine membranes contained 0.49 ± 0.16 N%, which served as the baseline for the elemental analysis of the crosslinked membranes. 

To increase the chemical stability, the as-spun PS-PCMS blend membranes were chemically crosslinked using DMEDA at an increasing CMS:DMEDA ratio of 1:10, 1:48, and 1:97. SEM images were collected to assess changes in the membrane morphology as a result of the crosslinking reaction. The fiber diameters were 4.0 ± 2.6, 4.7 ± 1.4, 3.7 ± 2.2, and 4.5 ± 2.3 μm for the 1:0, 1:10, 1:48, and 1:97 crosslinking ratios, respectively. While there was no trend in the fiber diameter as a function of the CMS:DMEDA ratio, all membranes generally maintained a smooth, ribbon-like structure, as shown in Figure 1.

Elemental analysis was conducted to quantify the nitrogen content, and, therefore, the DMEDA content, of the membranes before and after crosslinking, as shown in Figure 1. The mean %N for the membranes crosslinked with the increasing CMS:DMEDA ratio (1:0, 1:10, 1:48, and 1:97) were found to be: 0.49 ± 0.16 wt%, 0.34 ± 0.11 wt%, 0.52 ± 0.11 wt%, and 0.64 ± 0.13 wt%. While the mean value of the %N increased with increasing DMEDA, statistical analysis showed that it was not significantly different. This result may be due to limitations of using elemental analysis at such a low nitrogen content. While an elemental analysis is commonly used in organic chemistry, analyzing porous and non-uniform samples, like membranes, is subject to higher errors [38]. Furthermore, the non-zero N content of the PS-PCMS polymer complicates the quantitative comparison. We speculate that the N content of the PS-PCMS samples is related to the polymer manufacturing methods. If N-containing polymerization initiators or solvents were used during the original synthesis of the polymers, these residual species could provide a N signal. This will be discussed further in the context of chemical stability below.

**Figure 2:**
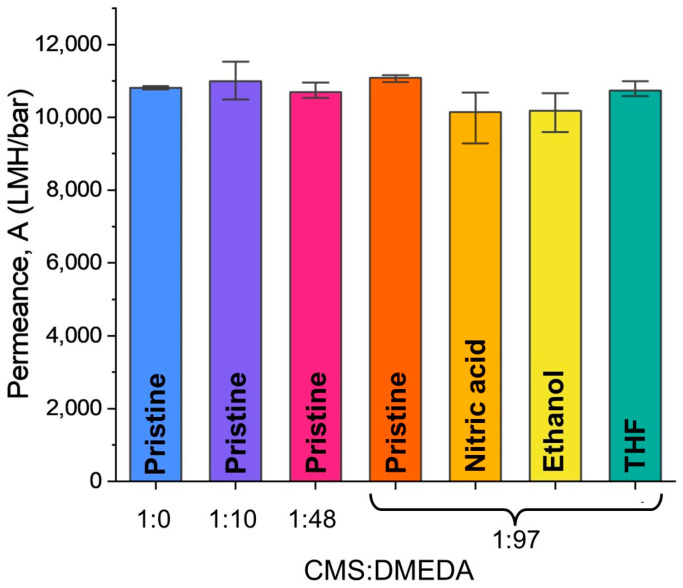
Permeance for PS-PCMS blend membranes prior to chemical testing (first four bars from the left). The last three bars represent the permeance of the 1:97 (CMS:DMEDA) crosslinked membranes after chemical resistance testing. Error bars represent the standard deviation.

Porosity (ε) is nominally unchanged with an increase in the CMS:DMEDA ratio (83.9 ± 3.6% to 81.5 ± 10.5%), and ANOVA testing supports that there is no statistical difference between the porosities of the crosslinked membranes. Furthermore, the SEM images depict similar morphological characteristics between the pristine, uncrosslinked and pristine, crosslinked membranes, supporting that the crosslinking does not impact the membrane morphology. The membrane permeance followed a similar trend. Pristine, uncrosslinked PS-PCMS blend membranes had a high permeance (A = 10,810 ± 48 LMH-bar^−1^), as shown in Figure 2, and there was no statistical difference between the permeance of pristine, uncrosslinked membranes and the pristine, crosslinked membranes at all crosslinking ratios. Thus, the crosslinking process did not impact the membrane porosity or permeance for the PS-PCMS blend membranes.

The chemical stability of the pristine and crosslinked membranes was evaluated via exposure to ethanol, 10 M HNO_3_, and THF. The results are summarized in Figure 1. When pristine, uncrosslinked membranes were exposed to ethanol, the membrane porosity decreased from 83.9 ±3.6% to 76.4 ± 6.0%. For all crosslinked membranes, the porosities after ethanol exposure were statistically lower than those before exposure. Despite the decrease, the porosity remained high—ranging from 78 ± 5% for the pristine membrane to 88 ± 1% for the 1:97 CMS:DMEDA sample. These measurements are supported by the SEM images, which show a smooth ribbon-like morphology with an interconnected structure for both pristine and crosslinked samples after exposure to ethanol. As seen in Figure 2, the permeance of the 1:97 CMS:DMEDA membrane before ethanol exposure (A = 11,080 ± 104 LMH-bar^−1^) is not statistically different than the permeance of the membrane after ethanol exposure (A = 10,180 ± 541 LMH-bar^−1^). Importantly, the individual permeance data showed a linear dependence of flux on transmembrane pressure—indicating that no membrane compaction occurred during filtration. These observations support the mechanical stability of the membranes under the tested conditions.

After exposure to 10 M HNO_3_, the porosities of the PS-PCMS blend membranes were not statistically different from the corresponding pre-exposure porosities. For example, the pristine, uncrosslinked membranes had a porosity of 85 ± 3% before nitric acid exposure and a porosity of 76.1 ± 3.9% after exposure. The 1:97 CMS:DMEDA membrane had a porosity of 81.5 ±10.5% before exposure and 85.4 ± 0.6% after nitric acid exposure. The remaining porosities are summarized in Figure 1. Similarly, the difference in permeance of the 1:97 CMS:DMEDA membrane before (A = 11,080 ± 104 LMH/bar) and after (A = 10,140 ± 747 LMH/bar) nitric acid exposure is not statistically significant, as shown in Figure 2.

The most challenging solvent was THF because the PS and PCMS polymers were originally dissolved in THF for electrospinning—so their solubilization is expected. Exposing the pristine, uncrosslinked membrane and the 1:10 CMS:DMEDA membrane to THF resulted in the full dissolution of the membrane. For this reason, there were no SEM images, porosity data, or permeance data to report. While the 1:48 and 1:97 CMS:DMEDA membranes did not dissolve, the porosities were statistically different from the pristine membranes—decreasing to 41.3 ± 1% and 47.0 ± 0.9%, respectively. These data are supported by the SEM images, which show visible compaction of the membranes, as shown in Figure 1. Based on the SEM and porosity data, it would seem that the degrees of crosslinking for the 1:48 and 1:97 CMS:DMEDA membranes were, in fact, higher than the uncrosslinked and 1:10 CMS:DMEDA membranes. Thus, we recognize the limitations of using the elemental analysis of N as a proxy for the degree of crosslinking in these samples. It is possible that the sensitivity of the analysis is not high enough to capture the true changes in the degree of crosslinking—as evidenced by their reproducible SEM images and permeance measurements after THF exposure. Surprisingly, the THF exposed membranes maintained a high permeance after exposure to THF, as shown in Figure 2. The 1:97 CMS:DMEDA membrane permeance before (A = 11,080 ± 104 LMH/bar) and that after THF exposure (A = 10,740 ± 218 LMH/bar) are not statistically different. Overall, the permeance of the PS-PCMS blend membranes that were crosslinked using a 1:97 CMS:DMEDA mass ratio was not impacted by exposure to the tested solvents (ethanol, THF, 10 M HNO_3_) and is a viable option for functionalization reactions that require exposure to organic solvents.

As stated in Equation (3), membrane permeance depends on the membrane thickness, tortuosity, porosity, and pore diameter. In this work, we directly measured membrane permeance and porosity. Membrane thickness was calculated from the filtration area, membrane mass, polymer density, polymer composition, and porosity. Example calculations are described in the Supporting Information. PS-PCMS membrane thickness did not change after the crosslinking reactions, as shown in Table 1. Together with the porosity and permeance measurements, these additional data suggest that the membrane morphology did not change before and after crosslinking with DMEDA.

Membrane thickness was calculated after solvent exposure experiments and is summarized in Table 2. Here, only the highest crosslinked PS-PCMS membranes were exposed to ethanol, 10 M HNO_3_, and THF. The membrane thickness notably decreases after exposure to ethanol and THF. The compaction of the membrane is evident from the decrease in porosity; however, the permeance remains unchanged. Thus, it is likely that the pore diameter and/or tortuosity change upon exposure to ethanol and THF. For this reason, the PS-PCMS membranes may be unsuitable for filtration applications or functionalization reactions that require contact with short-chain alcohols or THF. Pore size was not measured directly as these membranes have large, ill-defined three-dimensional “pores” that are highly interconnected. Thus, it is challenging to decouple pore diameter and tortuosity in these membranes.

### 3.2. PCMS Membranes

**Figure 3:**
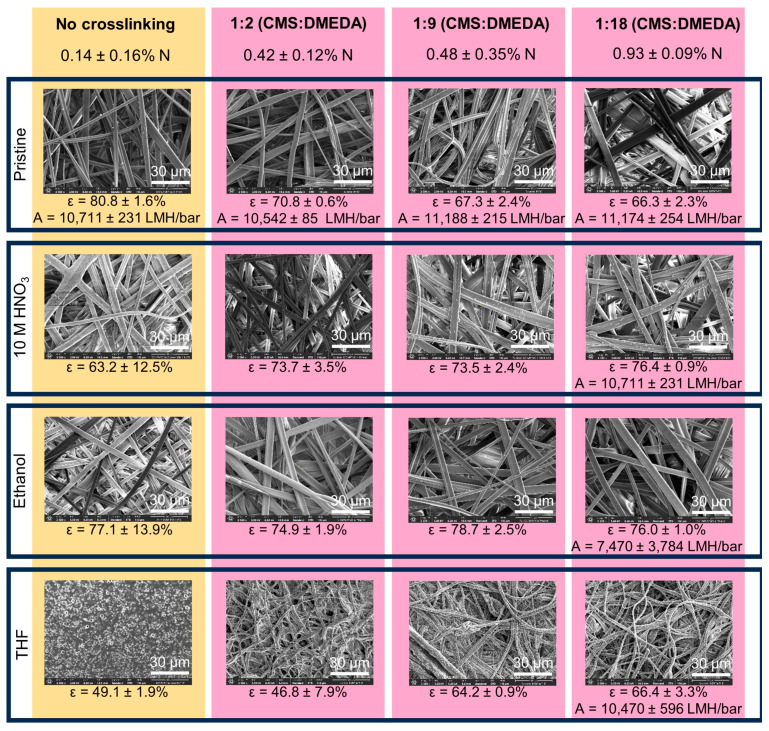
Data summary for PCMS membranes with representative SEM images of electrospun membranes with varying degrees of crosslinking (columns) exposed to different solvents (rows). Nitrogen content (N) and porosity (ε) were measured for all membranes. Permeance (A) was measured for all pristine membranes and 1:18 CMS:DMEDA membranes after each solvent exposure. Increasing the degree of crosslinking was qualitatively seen to increase the chemical stability of the fibers.

A second set of membranes were synthesized using high-molecular-weight PCMS (500,000 M_W_) and were characterized according to the same methods as the PS-PCMS blends. SEM images, porosity, and membrane permeance are summarized in Figure 3. Membranes were successfully synthesized, and the resulting fibers had an average fiber diameter of 1.8 ± 2.8 μm and porosity of 80.8 ± 1.6%, for the pristine, uncrosslinked membrane. The pristine, uncrosslinked membrane contained 0.14 ± 0.16 wt% N. This baseline N content is lower than the PS-PCMS blend, which enables a more reliable baseline for comparing N content in crosslinked samples. The permeance of the pristine, uncross linked membranes was 10,710 ± 230 LMH/bar, which is comparable to the PS-PCMS blend membrane (10,810 ± 48 LMH/bar).

Like the PS-PCMS blend membranes, the spun fibers were chemically crosslinked using DMEDA at an increasing CMS:DMEDA ratio of 1:2, 1:9, and 1:18. As shown in Figure 3, the SEM images depict similar morphological characteristics between the pristine and crosslinked membranes, suggesting that the crosslinking does not change the membrane morphology. The mean fiber diameter increased with the increasing DMEDA in the crosslinking reaction (1.8 ± 2.8 μm, 3.9 ± 2.4 μm, 5.3 ± 2.3 μm, 4.6 ± 2.3 μm) for the 1:0, 1:2, 1:9, and 1:18 crosslinking ratios, respectively; however, the diameters were not statistically different. All fiber diameter data are summarized in Table S1 in Supporting Information.

The mean nitrogen content increased (0.14 ± 0.16 wt%, 0.42 ± 0.12 wt%, 0.48 ± 0.35 wt%, and 0.93 ± 0.09 wt%,) with increasing CMS:DMEDA ratio of (1:0, 1:2, 1:9 and 1:18) during crosslinking, as measured by elemental analysis. Despite the apparent increase in the mean, only the 1:18 CMS:DMEDA sample demonstrated a statistically significant increase in N% over the pristine, uncrosslinked PCMS membrane. For the PCMS membranes, increasing the DMEDA concentration to 1:18 increased the nitrogen content—suggesting a higher degree of crosslinking. These estimates are further supported by the results of the chemical resistance tests, as discussed below.

**Figure 4:**
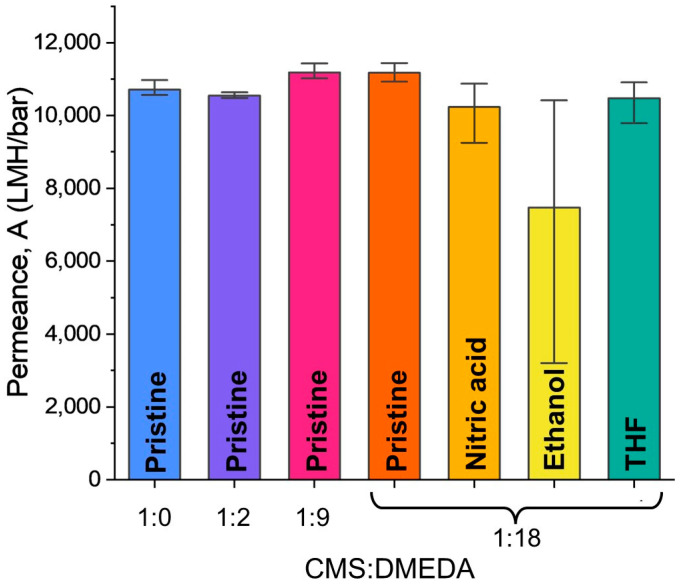
Permeance, A, for all PCMS membranes prior to chemical testing (first four bars from the left). The last 3 bars represent the permeance of the 1:18 crosslinked membranes after chemical resistance testing. Error bars represent the standard deviation.

The porosity (ε) of the membranes decreased from 80.8 ± 1.6% for the uncrosslinked, pristine membrane to 66.3 ± 2.3% for the 1:18 CMS:DMEDA membrane, and this decrease is statistically significant. Like the PS-PCMS blend membranes, the permeance for PCMS membranes remains high for all crosslinking conditions, as shown in Figure 4, despite the decrease in porosity. Similar to the PS-PCMS blend membranes, the permeance of the pristine, uncrosslinked PCMS membrane (A = 10,810 ± 48 LMH/bar) is not significantly different when compared to all of the crosslinked PCMS membranes.

The chemical stability of the pristine and crosslinked PCMS membranes was tested in ethanol, 10 M HNO_3_, and THF. The results are summarized in Figure 3. Ethanol exposure caused a statistically significant change in porosity when compared to the pristine membranes. At the highest degree of crosslinking (1:18 CMS:DMEDA), the porosity of the membrane before ethanol exposure was 66.3 ± 2.3%, while the porosity after ethanol exposure was 76 ± 1.0%. Although the porosity is different, the permeance is not statistically different between the 1:18 CMS:DMEDA membrane before exposure (A = 11,170 ± 254 LMH/bar) and the membrane (A = 7471 ± 3784 LMH/bar) after exposure, as shown in Figure 4.

PCMS membranes were exposed to 10 M HNO_3_, and no statistical differences were found for porosity or permeance before and after exposure. The porosities of the 1:18 CMS:DMEDA membranes before (66.3 ± 2.3%) and after (76.4 ± 0.9%) 10 M HNO_3_ exposure remained high. Similarly, the permeance of the 1:18 CMS:DMEDA membranes after 10 M HNO_3_ exposure (A = 10,230 ± 865 LMH/bar) was not statistically different from the permeance before exposure (A = 11,170 ± 254 LMH/bar), as shown in Figure 4.

Finally, the stability of the PCMS membranes was tested in THF. Unlike the PS-PCMS blend membranes, none of the PCMS membranes fully dissolved in THF. SEM images of the uncrosslinked PCMS membrane in THF show clear morphological changes in the membrane, including pore collapse and apparent dissolution of the fibers. The characteristic pore structure of the uncrosslinked, electrospun membrane was lost after THF exposure and is reflected in the low porosity (49.1 ± 1.9%) of the membrane. For the samples with higher degrees of crosslinking, like the 1:18 CMS:DMEDA membranes, the porosity (66.4 ± 3.3%) is not statistically different than the pristine, crosslinked membrane before THF exposure (66.3 ± 2.3%). The permeance was measured for the 1:18 CMS:DMEDA membranes before and after THF exposure, as shown in Figure 4, and no statistical differences were found. For example, the permeance of the 1:18 CMS:DMEDA membranes is A = 11,170 ± 254 LMH/bar and the permeance of the same membranes after THF exposure is A = 10,470 ± 596 LMH/bar.

As stated previously, membrane thickness was calculated from the filtration area, membrane mass, polymer density, polymer composition, and porosity (see Supporting Information). PCMS membrane thickness did not change after the crosslinking reactions, as shown in Table 3. Thus, for both membrane designs (PS-PCMS and PCMS), the DMEDA crosslinking reaction does not impact membrane morphology (fiber diameter, porosity, thickness).

Membrane thickness was calculated after solvent exposure experiments (ethanol, 10 M HNO_3_, and THF) and is summarized in Table 4. Only the highest crosslinked PS-PCMS membranes were tested, and, notably, there was no decrease in membrane thickness. Thus, the PCMS membranes exhibit no morphological changes (fiber diameter, porosity, thickness) after exposure to the tested solvents. For this reason, the PCMS membranes may be suitable for filtration applications or functionalization reactions that require contact with short-chain alcohols or THF. Further investigations will be required to assess the synthesized membranes in application-specific size exclusion experiments or adsorption experiments after functionalization.

## 4. Discussion

Membranes were successfully electrospun from PS-PCMS blend and PCMS polymer dope solutions. The resulting membranes had porosities > 80% and permeances of ~10,000 LMH/bar, and they contained defect-free fibers with diameters on the order of 2–5 microns. Overall, the uncrosslinked PCMS membranes were more chemically stable than the uncrosslinked PS-PCMS blend membranes, as the PCMS membranes did not dissolve in THF. This is likely due to the higher molecular weight of the PCMS polymer (M_w_ 500,000 g/mol) as compared to the PS-PCMS blend (M_w_ < 350,000 g/mol). All uncrosslinked membranes showed no change in porosity or fiber diameter after exposure to ethanol and 10 M HNO_3_. Membrane stability in nitric acid is of particular importance to the field of isotope production and radioanalytical chemistry because separations are commonly performed in strong acids. Thus, these electrospun membranes have the requisite acid stability for radiochemistry applications as filtration times are typically <24 h.

PCMS and PS-PCMS blend membranes were crosslinked using DMEDA with the goal of increasing the chemical stability, though the degree of crosslinking was challenging to quantify through an elemental analysis. For the three crosslinking conditions tested, only the 1:18 (CMS:DMEDA) reaction resulted in a statistically significant increase in N% in the PCMS membranes. As noted in Scheme 1, the presence of N does not guarantee crosslinking—but the increased chemical stability supports the notion that crosslinking occurred. For both PS-PCMS and PCMS membranes, the crosslinking reactions did not impact the fiber diameter, porosity, membrane thickness, or permeance.

For all PS-PCMS blend and PCMS membranes at the highest CMS:DMEDA ratio, the permeance did not change after solvent exposure, remained high (>10,000 LMH/bar), and was consistent with other commercially available, chemically robust membranes like PVDF. For the PS-PCMS blend membranes with the highest CMS:DMEDA ratio, the permeance did not change after exposure to ethanol, 10 M nitric acid, or THF; however, the membranes did experience some morphological changes. After exposure to ethanol and THF, the membranes compacted and exhibited a decrease in porosity and thickness. It is possible that additional morphological changes such as pore diameter and/or tortuosity occurred during solvent exposure. Thus, the highly crosslinked PS-PCMS membranes are not chemically stable enough to use for filtration or functionalization experiments that require alcohols or organic solvents.

The PCMS membranes crosslinked with the highest CMS:DMEDA ratio did not exhibit morphological changes (porosity, fiber diameter, membrane thickness) after solvent exposure and maintained a high permeance. Thus, chemically cross-linking PCMS electrospun membranes is a viable technique for increasing the chemical stability of the membranes. An additional practical implication is that the PCMS membranes crosslinked with the highest CMS:DMEDA ratio can be chemically processed (i.e., exposed to organic solvents) for at least 24 h without impacting membrane permeance—opening the door for future functionalization reactions such as AGET ATRP, RAFT polymerization, and S_N_2 reactions, which can be performed at the chloromethyl moiety of the PCMS in the fibers.

## Figures and Tables

**Table 1:** PS-PCMS membrane thickness before and after crosslinking reactions using DMEDA.

Membrane	No Crosslinking	Crosslinked Membranes		
		CMS:DMEDA (1:10)	CMS:DMEDA (1:48)	CMS:DMEDA (1:97)
PS-PCMS	0.776 ± 0.106 mm	1.075 ± 0.079 mm	0.832 ± 0.076 mm	0.973 ± 0.155 mm

**Table 2:** PS-PCMS membrane thickness before and after solvent exposure for the 1:97 CMS:DMEDA membranes.

Crosslinked Membrane (CMS:DMEDA)	No Solvent	Solvent-Exposed Membranes		
		Ethanol	10 M HNO_3_	THF
PS-PCMS (1:97)	0.973 ± 0.155 mm	0.533 ± 0.060 mm	1.248 ± 0.088 mm	0.268 ± 0.031 mm

**Table 3:** PCMS membrane thickness before and after crosslinking reactions using DMEDA.

Membrane	No Crosslinking	Crosslinked Membranes		
		CMS:DMEDA (1:2)	CMS:DMEDA (1:9)	CMS:DMEDA (1:18)
PCMS	0.651 ± 0.025 mm	0.529 ± 0.094 mm	0.514 ± 0.057 mm	0.485 ± 0.068 mm

**Table 4:** PCMS membrane thickness before and after solvent exposure for the 1:18 CMS:DMEDA membranes.

Crosslinked Membrane (CMS:DMEDA)	No Solvent	Solvent-Exposed Membranes		
		Ethanol	10 M HNO_3_	THF
PCMS (1:18)	0.4849 ± 0.0675 mm	0.5696 ± 0.0186 mm	0.6933 ± 0.1371 mm	0.5250 ± 0.0605 mm

## Data Availability

The data that support the findings of this study are openly available on the Harvard Dataverse at https://doi.org/10.7910/DVN/MCJ5SP.

## Supplementary Materials

The following supporting information can be downloaded at: https://www.mdpi.com/article/10.3390/membranes13110870/s1, Electrospinning apparatus diagram (Figures S1 and S2), electrospinning system parts list (Table S1), fiber diameter (Table S2), statistical analyses (Figures S3–S17), calculation of membrane thickness.
