# Supplementary material for: Chemically Stable Styrenic Electrospun Membranes with Tailorable Surface Chemistry
Source: Membranes (Basel). 2023 Nov 2;13(11):870. doi: 10.3390/membranes13110870 (PMC10673432; doi:10.3390/membranes13110870)
Supplement: Supplementary file 1 [file membranes-13-00870-s001.zip › membranes-2670124-supplementary.pdf]

Supporting Information

for

Chemically-stable styrenic electrospun membranes with tailorable surface chemistry

Maura Sepesy[§], Tuli Banik[§], Joelle Scott, Luke A.F. Venturina, Alec Johnson, Bernadette L. Schneider, Megan M. Sibley, and Christine E. Duval*

Department of Chemical and Biomolecular Engineering, Case Western Reserve University, 10900 Euclid Avenue, Cleveland, OH 44106

[§]Co-first authors

*Corresponding author. Current address: Department of Chemical and Biomolecular Engineering, Case Western Reserve University, 10900 Euclid Avenue, Cleveland, OH 44106, USA. Tel: +1 (216) 368-8613, Email: christine.duval@case.edu

Keywords

Microfiltration, adsorptive chromatography, surface functionalization, non-woven membranes

Table of Contents

1. Construction of the Electrospinning System 3
2. Electrospinning apparatus 4
3. List of parts required to build the electrospinning system 5
4. Fiber Diameter Data, Table S2 6
5. Statistics 7
  5.1 Normality tests 7
  5.2 One-way Analysis of Variance (ANOVA) 14
    5.2.1 Permeance for 80/20 blend given different crosslinking ratios and no chemical testing 14
    5.2.2 Permeance for 80/20 blend given 1:10 crosslinking and chemical testing 15
    5.2.3 Porosity for 80/20 blend given different crosslinking ratios and no chemical testing 16
    5.3.4 Porosity for 80/20 blend given 1:10 crosslinking and ethanol chemical testing 17
    5.2.5 Permeance for PCMS given different crosslinking ratios and no chemical testing 18
    5.2.6 Porosity for PCMS given different crosslinking ratios and no chemical testing 19
    5.2.7 Porosity for PCMS given different crosslinking ratios and ethanol chemical testing 20
    5.2.8 Porosity for PCMS given different crosslinking ratios and nitric acid chemical testing 21
    5.2.9 %N for 80/20 blend given different crosslinking ratios and no chemical testing 22

5.2.10 %N for PCMS given different crosslinking ratios and no chemical testing................23
5.3 Mann-Whitney test 24
5.3.1 Porosity for 80/20 blend given 1:10 crosslinking and nitric acid chemical testing.......24
5.3.2 Porosity for 80/20 blend given 1:10 crosslinking and THF chemical testing...............25
5.3.3 Permeance for PCMS given 1:10 crosslinking and ethanol chemical testing.............25
5.3.4 Permeance for PCMS given 1:10 crosslinking and nitric acid chemical testing 26
5.3.5 Permeance for PCMS given 1:10 crosslinking and THF chemical testing..................27
5.3.6 Porosity for PCMS given different crosslinking ratios and THF chemical testing 27
5.3.7 Fiber diameter for PCMS membranes given pristine and 1:1 crosslinking ratio 28
5.3.8 Fiber diameter for PCMS membranes given pristine and 1:5 crosslinking ratio 29
5.3.9 Fiber diameter for PCMS membranes given pristine and 1:10 crosslinking ratio 29
5.3.10 Fiber diameter for PCMS membranes given 1:1 and 1:5 crosslinking ratio..............30
5.3.11 Fiber diameter for PCMS membranes given 1:1 and 1:10 crosslinking ratio............31
5.3.12 Fiber diameter for PCMS membranes given 1:5 and 1:10 crosslinking ratio............31
5.3.13 Fiber diameter for PCMS and Blend membranes 32
5.3.14 Fiber diameter for Blend membranes given pristine and 1:1 crosslinking ratio 33
5.3.15 Fiber diameter for Blend membranes given pristine and 1:5 crosslinking ratio 33
5.3.16 Fiber diameter for Blend membranes given pristine and 1:10 crosslinking ratio 34
5.3.17 Fiber diameter for Blend membranes given 1:1 and 1:5 crosslinking ratio...............35
5.3.18 Fiber diameter for Blend membranes given 1:1 and 1:10 crosslinking ratio.............35
5.3.19 Fiber diameter for Blend membranes given 1:5 and 1:10 crosslinking ratio.............36
6.0 Calculation of membrane thickness 37

1. Construction of the Electrospinning System

The following sections contains the detailed description of the electrochemical system that was built for this work. An additional diagram is shown in **Section 2** of the SI and the complete parts list is shown in **Section 3** of the SI.

1.1 Static box dimensions and construction

The electrospinning apparatus is contained within an acrylic static box. The overall dimensions of the box are 90 cm long, 51 cm wide, and 53 cm tall. There are cabinet-style doors that open to the front (Diagram in Supporting Information, **Figure S1**) and the box is separated into two compartments using a sheet of 0.5 cm thick acrylic. This separator acts as a static shield to deter the static charge from arcing between the syringe pump and the power supply. On right wall of the static box, a 12.5 cm diameter hole was cut with a 1.5 cm lip to attach a rubber glovebox glove.

1.2 Electrospinning hardware

Within the left-hand compartment of the static box, the syringe pump (NE-1000 programmable single syringe pump, New Era Pumps) is placed on a laboratory jack wrapped with electrical tape. To supply power to the syringe pump, an extension cord was cut, threaded through a hole in the back of the static box and rewired with an electrical plug (female). A hole was drilled in the lower right corner to feed a 250V extension cord with new electrical plug (female) to power the power supply (CHARGEMASTER VCM60-P STD DIS). A [4-in-1] 12AWG 15-amp household AC plug to 20 Amp T blade adapter cable is used to plug the 250V extension cord into the wall outlet. The system was grounded using a 10 gauge insulated ground wire. One end of the wire was connected to an electrical plug (male) and the other end was affixed to a screw protruding from the back of the static box. The electrical plug of the ground wire is plugged into a standard wall outlet to ground the system. On the right-hand side of the partition, the grounded collector plate is prepared by wrapping an 20.3 x 20.3 cm aluminum plate that is 1.9 cm thick in aluminum foil. Then the wrapped plate is placed on an adjustable, non-conducting polycarbonate lab jack (POLY-JAQUE).

1.3 Humidity Control System

In the right-hand compartment which contains the collector plate, a hole was drilled to connect the humidifier tubing to the box. The humidifier system (BaoGuai reptile mister fogger/humidifier) is located outside of the static box and humidified air is delivered to the spinning compartment via hose. An acrylic basket (wall-mount clear file holder tape-on/screw-in mounting with 3-3/4" wide compartment, McMaster-Carr) was installed using acrylic adhesive to hold the humidity sensors. One sensor is integrated with the humidity controller (WILLHI WH1436H 110V digital air humidity controller with LCD Display). The second sensor serves as an additional reference point (Fisherbrand™ traceable™ alarm RH/temperature monitor).

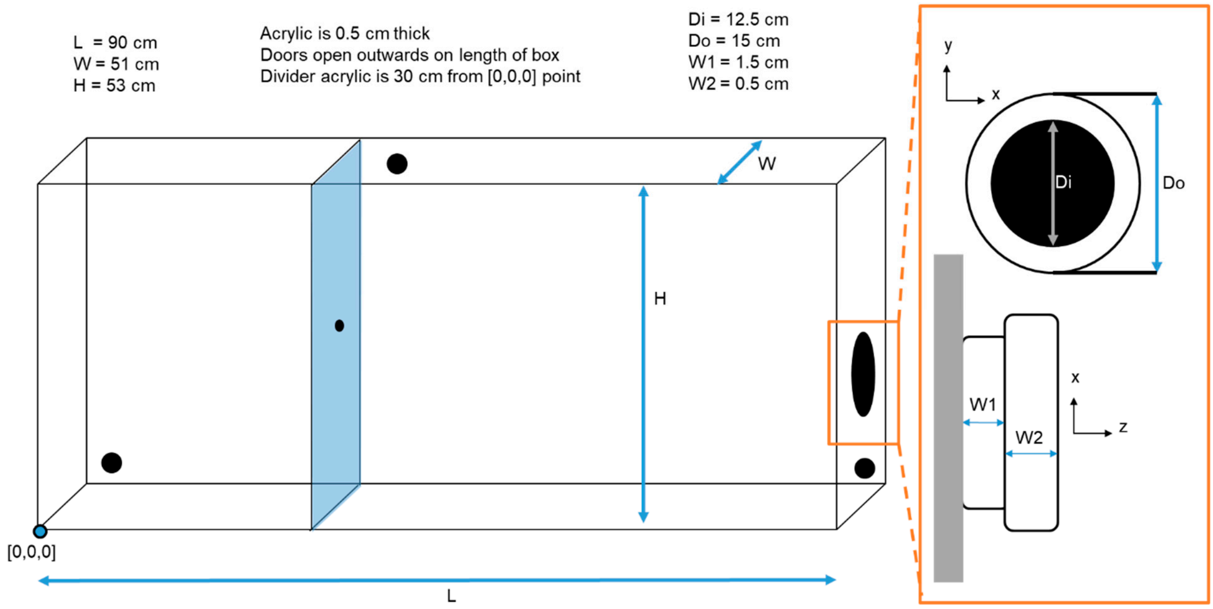

Figure S1: Model of the acrylic static box built to house the electrospinning apparatus. Not shown are the doors that open outwards. The three holes on the back bottom left, right and top middle are inlets for the power supplies and humidifier. The blue highlighted sheet in the middle of the box is the acrylic divider, separating the syringe pump from the collector plate and power supply. There is a hole to thread the 22-gauge needle through. The hole on the left side of the box is an inlet where a rubber glove can be attached to operate the power supply in the box.

2. Electrospinning apparatus

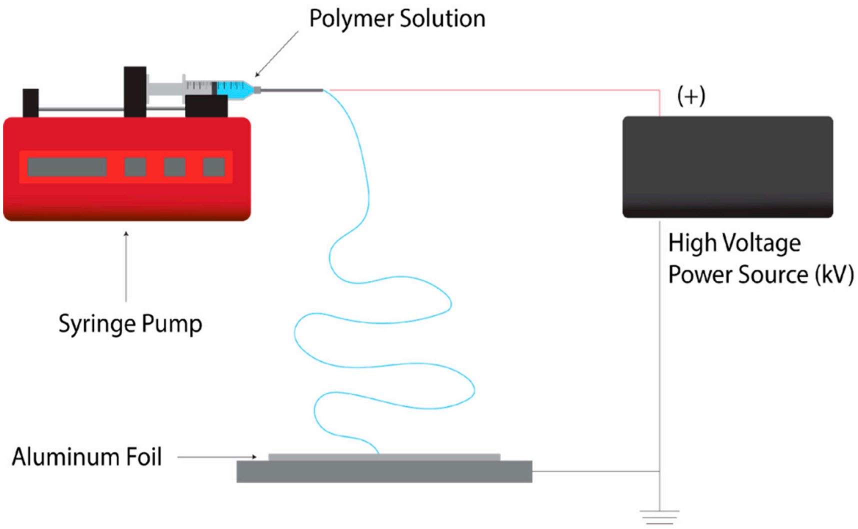

Figure S2: Electrospinning apparatus. An Al plate is covered in Al foil and grounded. The high voltage power supply is connected to the ground for safety, and applies a voltage to the needle of the syringe located on the syringe pump. This difference in electrical potential drives the polymer in the needles to the grounded plate.

3. List of parts required to build the electrospinning system

Table S1: List of parts required to build the electrospinning system

Item	CAT #	Supplier	Unit	Cost/unit	Total
Acrylic box	N/A custom build	House of Plastics	1	$ 762.33	$ 762.33
NE-1000 Programmable Single Syringe Pump	NE-1000	New Era	1	$ 775.00	$ 775.00
Ultra-Flexible Grounding Wire Insulated, 10 GaugeLength	2196K56	McMaster Carr	5	$ 1.10	$ 5.50
CHARGEMASTER VCM60-N STD DIS	4012559	SIMCO ION	1	$3,631.00	$3,631.00
aluminum 8"x8" sheet 1/4" thick	9246K11	McMaster Carr	1	$ 18.31	$ 18.31
Alligator Clip High-Current, Toothed, 304 Stainless Steel, 30 A, Black		McMaster Carr	1	$ 2.72	$ 2.72
Alligator Clip High-Current, Toothed, 304 Stainless Steel, 30 A, Red	7236K454	McMaster Carr	1	$ 2.72	$ 2.72
Female Disconnect Fully Insulated Nylon 12-10 AWG, .250 Series, 10/Clam		Tyco Electronics	1	$ 4.91	$ 4.91
POLY-JAQUE PLASTIC LAB JACK	F18391-0001	SP Bel-Art	1	$ 337.30	$ 337.30
Reptile Mister Fogger/Humidifier - Large Tank - Flexible Hose Suitable with Reptile Terrarium and Hide/Cave	B081LHQKZY	BaoGuai	1	$ 46.00	$ 46.00
WH1436H 110V Digital Air Humidity Controller with LCD Display High Accuracy Humidity Sensor & 118'' Power Cable, Humidification Mode & Dehumidification Mode	B01IJ8YWYY	WILLHI	1	$ 42.99	$ 42.99
9 ft. 12/3 SPT-3 3-Wire 20-Amp Air Conditioner/Major Appliance Power Cord		Southwire	1	$ 18.02	$ 18.02
Black Extension Cord 10 ft. Long, 0.44" OD	9581T16	McMaster Carr	1	$ 18.03	$ 18.03
12AWG 15 Amp Household AC Plug to 20 Amp T Blade Adapter Cable,5-15P to 5-20R,5-15P to 6-15R,5-15P to 6-20R, 4 in 1 AC Adapter,15A 125V to 20A 250V 15A 125V to 20A 250V Adapter Cable, 1FT	AUB08PNNMNC4	Plugrand	1	$ 11.99	$ 11.99
3-Slot Straight Socket, Grounded, NEMA 6-15	7196K42	McMaster Carr	1	$ 16.83	$ 16.83
Hinged Straight-Blade Housing Hinged, 3-Blade Plug, Grounded, NEMA 5-15, Yellow	7216K51	McMaster Carr	2	$ 9.77	$ 19.54
30 mL glass syringe	80089-556	VWR	6	$ 19.86	$ 119.16
SS Dispensing Needle with Luer Lock Conn 2" Needle Length, 22 Gauge, Packs of 50	75165A254	McMaster Carr	1	$ 15.05	$ 15.05
Wire cutter (24-14) and stripper (26-16)	7294K14	McMaster Carr	1	$ 15.62	$ 15.62

4. Fiber Diameter Data, Table S2

Table S1: Fiber diameter data for all PS-PCMS blend membranes and PCMS membranes. The crosslinking column represents the mass ratio of PCMS to DMEDA used during the crosslinking reaction.

Polymer	Crosslinking (PCMS:DMEDA)	Solvent Exposure	Fiber Diameter (μm)
PS-PCMS blend	1:0	None-pristine	4.0 ± 2.6
PS-PCMS blend	1:1	None-pristine	4.7 ± 1.4
PS-PCMS blend	1:5	None-pristine	3.7 ± 2.2
PS-PCMS blend	1:10	None-pristine	4.5 ± 2.3
PCMS	1:0	None-pristine	1.8 ± 2.8
PCMS	1:1	None-pristine	3.9 ± 2.4
PCMS	1:5	None-pristine	5.3 ± 2.3
PCMS	1:10	None-pristine	4.6 ± 2.3

5. Statistics

5.1 Normality tests

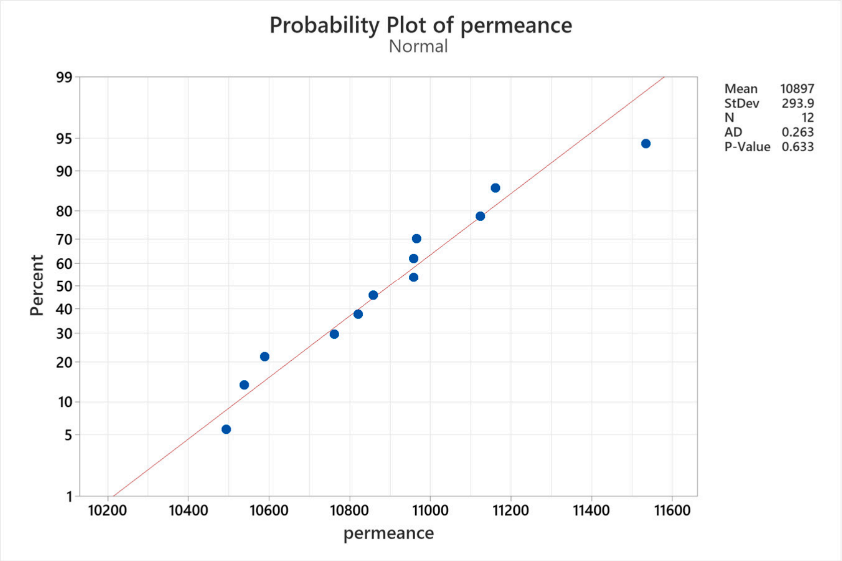

Figure S3: Probability plot of permeance for 80/20 crosslinking blends with no chemical testing; P-value > 0.05 thus data follows normal distribution

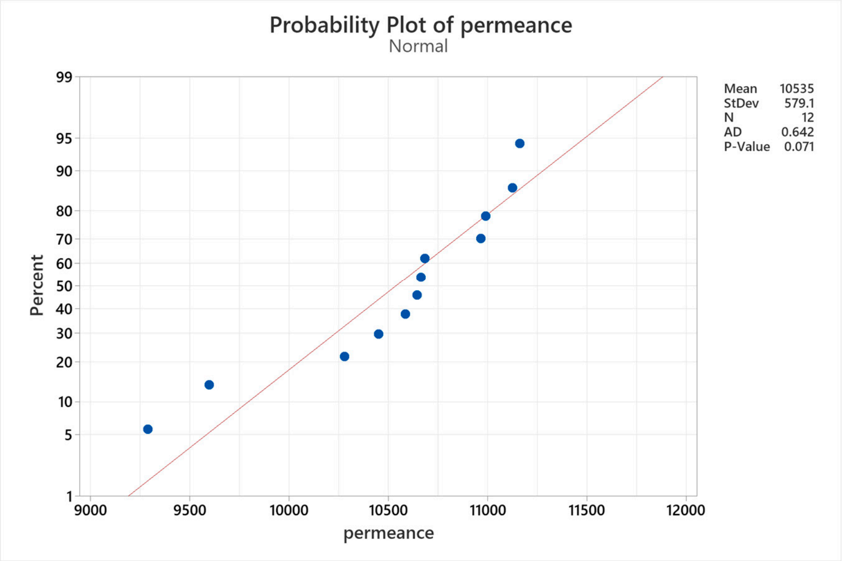

Figure S4: Probability plot of permeance for 80/20 with 1:10 crosslinking with chemical testing; P-value > 0.05 thus data follows normal distribution

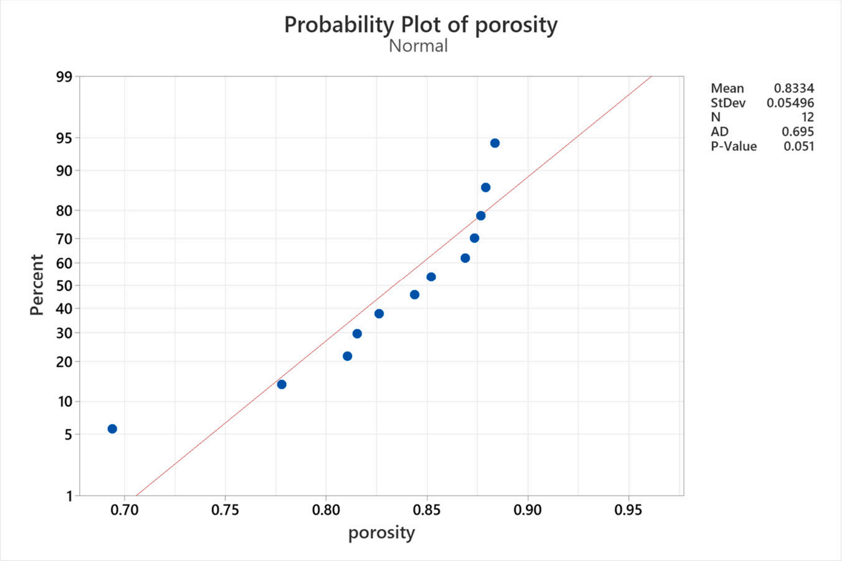

Figure S5: Probability plot of porosity for 80/20 crosslinking blends with no chemical testing; P-value > 0.05 thus data follows normal distribution

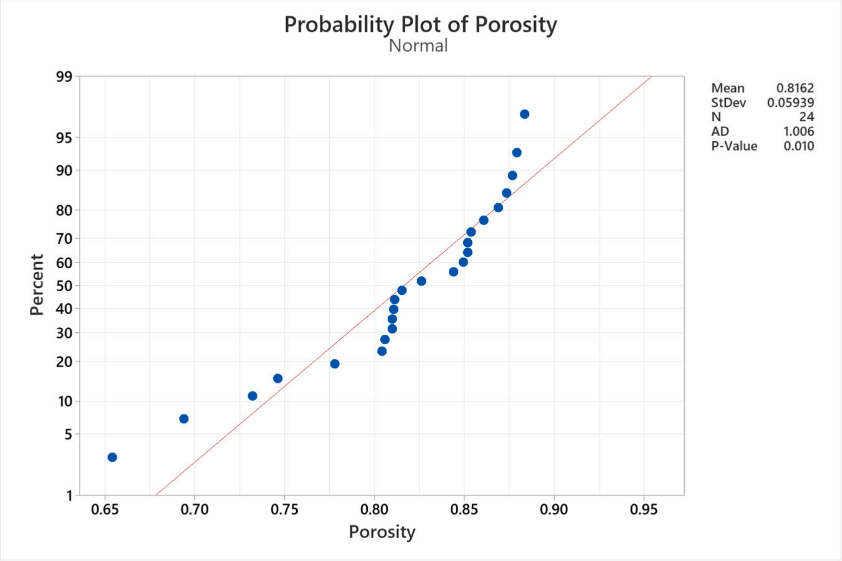

Figure S6: Probability plot of porosity for 80/20 with crosslinking blends with nitric acid chemical testing; P-value < 0.05 thus data does not follow a normal distribution

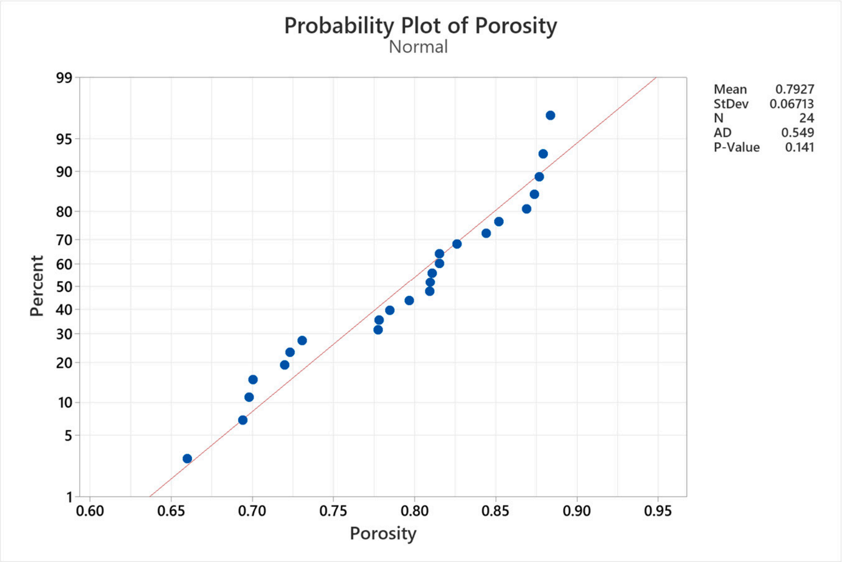

Figure S7: Probability plot of porosity for 80/20 with crosslinking blends with ethanol chemical testing; P-value > 0.05 thus data follows a normal distribution

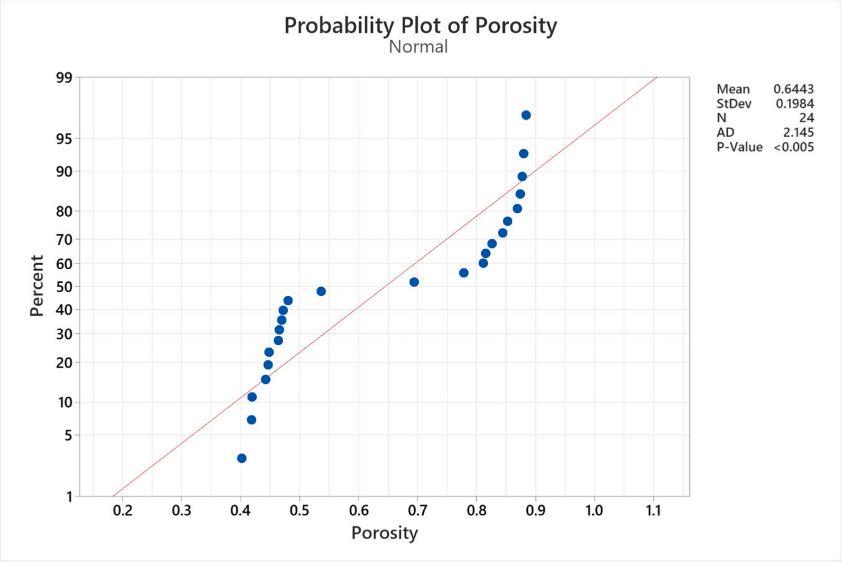

Figure S8: Probability plot of porosity for 80/20 with crosslinking blends with THF chemical testing; P-value < 0.05 thus data does not follow a normal distribution

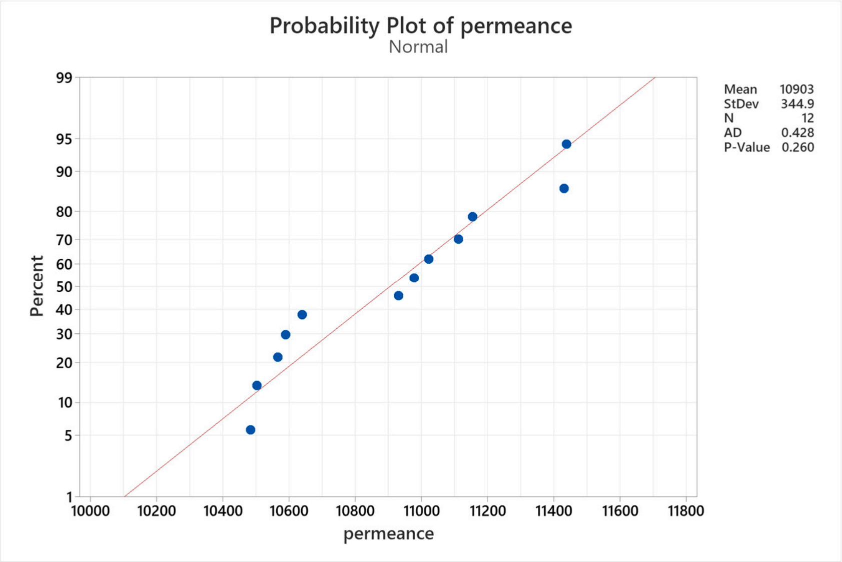

Figure S9: Probability plot of permeance for PCMS crosslinking blends with no chemical testing; P-value > 0.05 thus data follows normal distribution

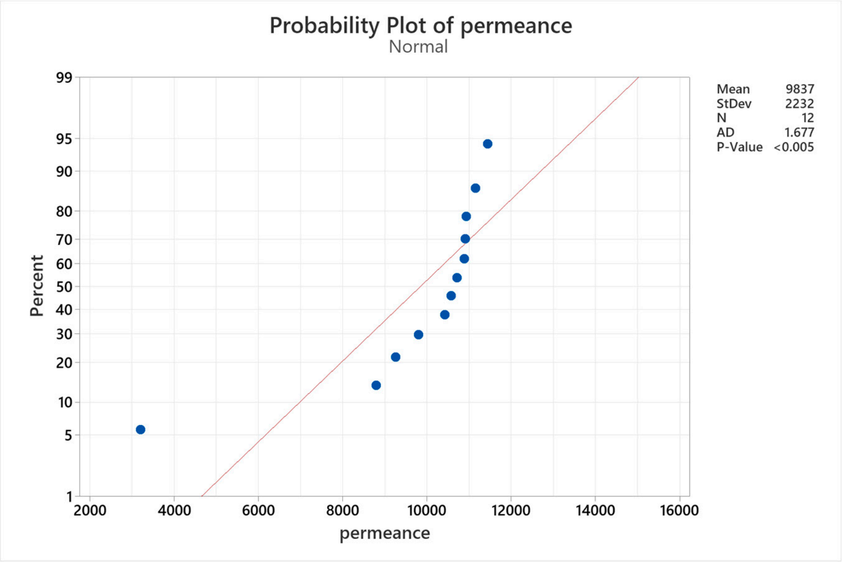

Figure S10: Probability plot of permeance for PCMS 1:10 crosslinking with chemical testing; P-value < 0.05 thus data does not follow normal distribution

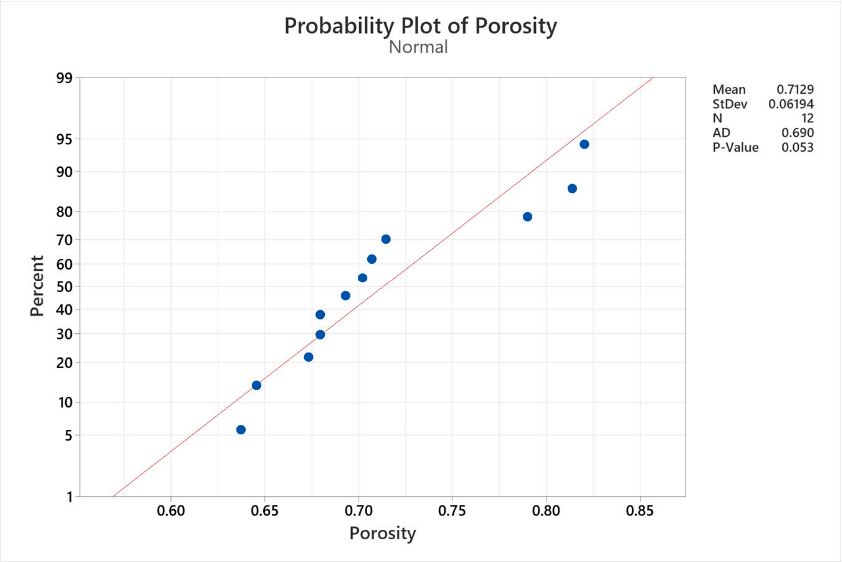

Figure S11: Probability plot of porosity for PCMS crosslinking blends with no chemical testing; P-value > 0.05 thus data follows normal distribution

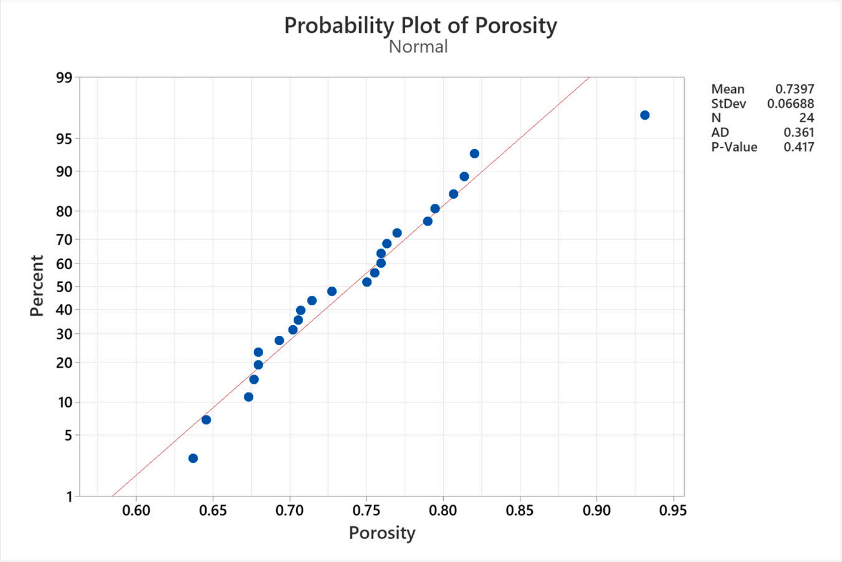

Figure S12: Probability plot of porosity for PCMS with crosslinking blends with ethanol chemical testing; P-value > 0.05 thus data follows a normal distribution

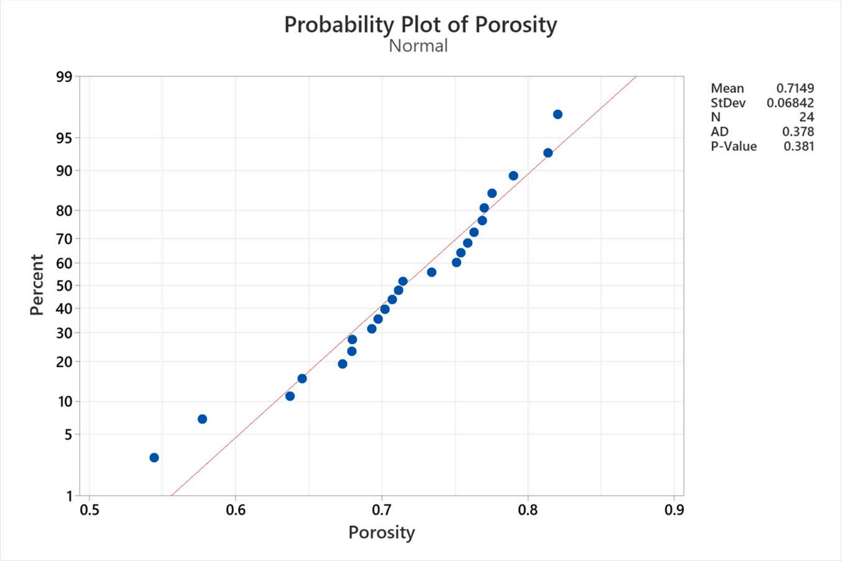

Figure S13: Probability plot of porosity for PCMS with crosslinking blends with nitric acid chemical testing; P-value > 0.05 thus data follows a normal distribution

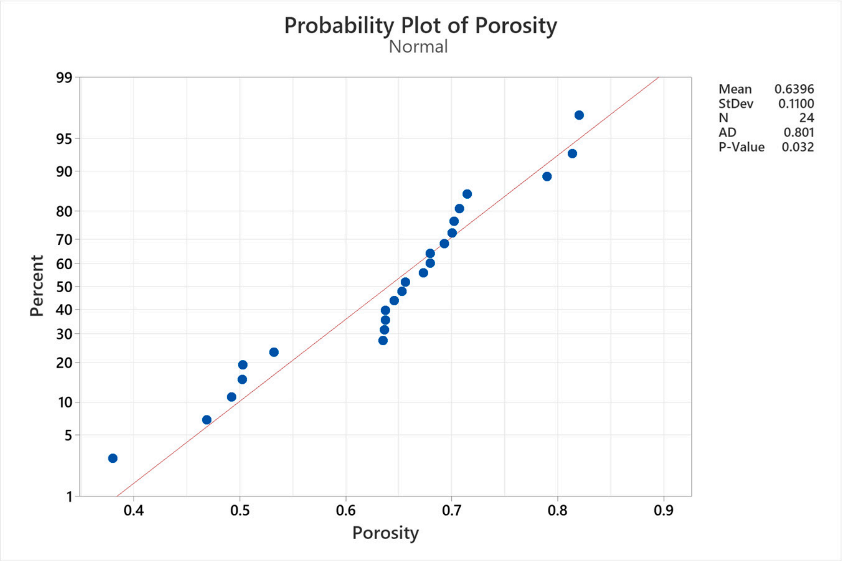

Figure S14: Probability plot of porosity for PCMS crosslinking blends with THF chemical testing; P-value < 0.05 thus data does not follow normal distribution

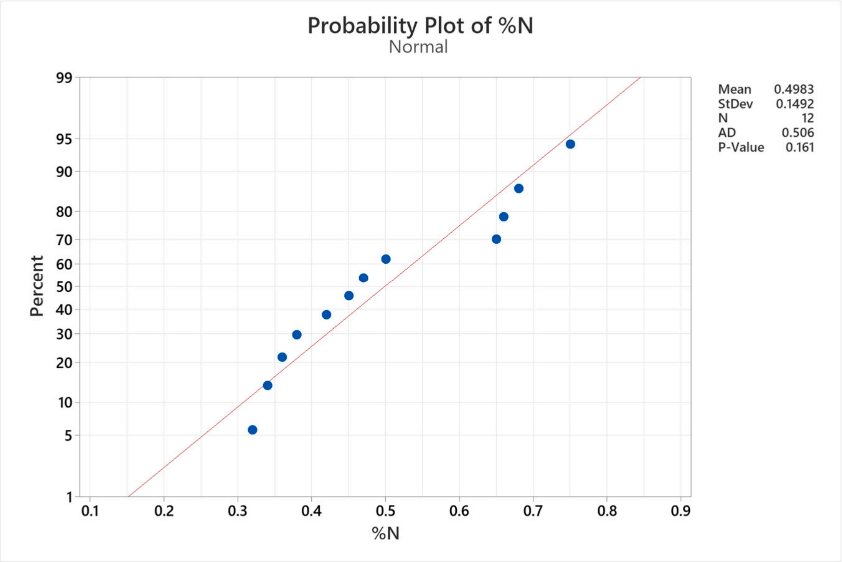

Figure S15: Probability plot %N for 80/20 blend with crosslinking blends; P-value > 0.05 thus data follows a normal distribution

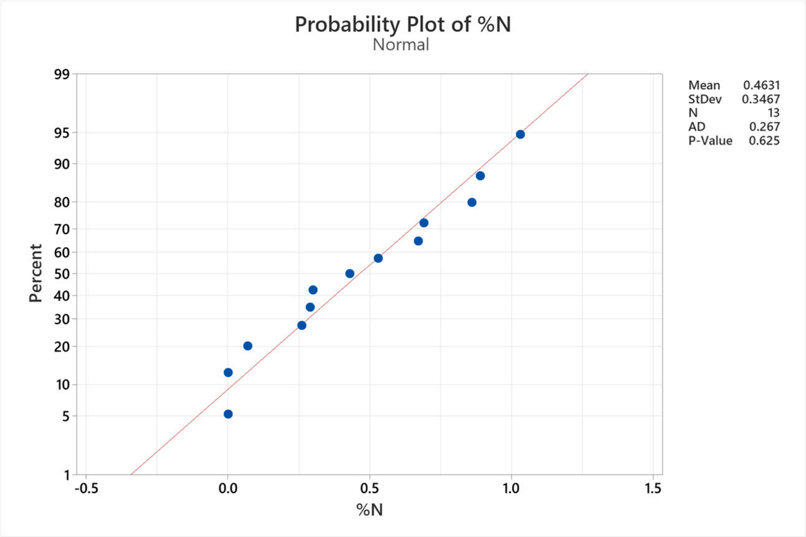

Figure S16: Probability plot %N for PCMS with crosslinking blends; P-value > 0.05 thus data follows a normal distribution

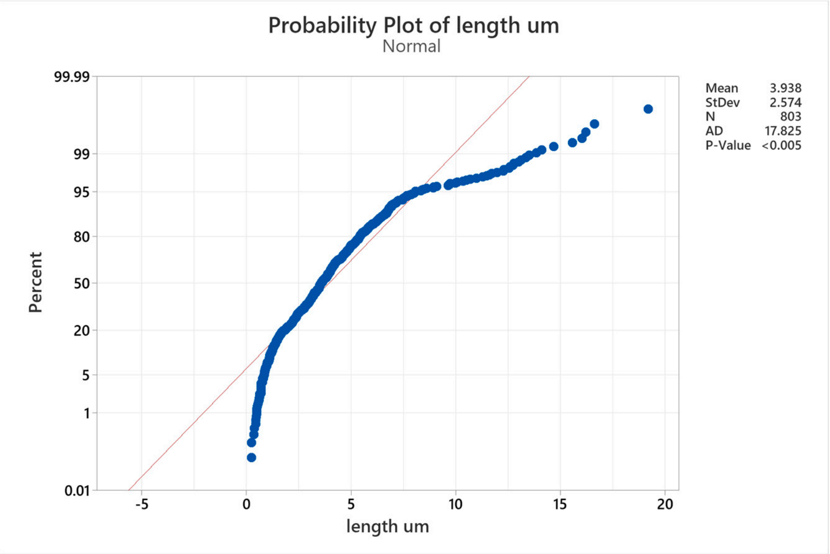

Figure S17: Probability plot of porosity for fiber diameters for the different polymer blends and crosslinking ratios; P-value < 0.05 thus data does not follow normal distribution

5.2 One-way Analysis of Variance (ANOVA)

5.2.1 Permeance for 80/20 blend given different crosslinking ratios and no chemical testing

Method

Null hypothesis	All means are equal
Alternative hypothesis	Not all means are equal
Significance level	α = 0.05

Equal variances were assumed for the analysis.

Factor Information

Factor	Levels	Values
membrane	4	pristine, x1, x10, x5

Analysis of Variance

Source	DF	Adj SS	Adj MS	F-Value	P-Value
membrane	3	275761	91920	1.09	0.407
Error	8	674311	84289		

Total	11	950072

Model Summary

S	R-sq	R-sq(adj)	R-sq(pred)
290.326	29.03%	2.41%	0.00%

Means

membrane	N	Mean	StDev	95% CI
pristine	3	10813.2	48.2	(10426.7, 11199.8)
x1	3	10995	521	(10608, 11381)
x10	3	11083.1	104.0	(10696.5, 11469.6)
x5	3	10695	229	(10309, 11082)

Pooled StDev = 290.326

Tukey Pairwise Comparisons

Grouping Information Using the Tukey Method and 95% Confidence

membrane	N	Mean	Grouping
x10	3	11083.1	A
x1	3	10995	A
pristine	3	10813.2	A
x5	3	10695	A

Means that do not share a letter are significantly different.

5.2.2 Permeance for 80/20 blend given 1:10 crosslinking and chemical testing

Method

Null hypothesis	All means are equal
Alternative hypothesis	Not all means are equal
Significance level	$\alpha = 0.05$

Equal variances were assumed for the analysis.

Factor Information

Factor	Levels	Values
chemical	4	ethanol, nitric, none, THF

Analysis of Variance

Source	DF	Adj SS	Adj MS	F-Value	P-Value
chemical	3	1872093	624031	2.75	0.112
Error	8	1816219	227027		

Total	11	3688311

Model Summary

S	R-sq	R-sq(adj)	R-sq(pred)
476.474	50.76%	32.29%	0.00%

Means

chemical	N	Mean	StDev	95% CI
ethanol	3	10179	541	(9545, 10813)
nitric	3	10141	747	(9506, 10775)
none	3	11083.1	104.0	(10448.7, 11717.4)
THF	3	10739	218	(10104, 11373)

Pooled StDev = 476.474

Tukey Pairwise Comparisons

Grouping Information Using the Tukey Method and 95% Confidence

chemical	N	Mean	Grouping
none	3	11083.1	A
THF	3	10739	A
ethanol	3	10179	A
nitric	3	10141	A

Means that do not share a letter are significantly different.

5.2.3 Porosity for 80/20 blend given different crosslinking ratios and no chemical testing

Method

Null hypothesis	All means are equal
Alternative hypothesis	Not all means are equal
Significance level	$\alpha = 0.05$

Equal variances were assumed for the analysis.

Factor Information

Factor	Levels	Values
membrane	4	1:1, 1:10, 1:5, pristine

Analysis of Variance

Source	DF	Adj SS	Adj MS	F-Value	P-Value
membrane	3	0.005833	0.001944	0.57	0.652
Error	8	0.027392	0.003424		

Total	11	0.033225

Model Summary

S	R-sq	R-sq(adj)	R-sq(pred)
0.0585153	17.55%	0.00%	0.00%

Means

membrane	N	Mean	StDev	95% CI
1:1	3	0.86731	0.01349	(0.78941, 0.94522)
1:10	3	0.8154	0.1055	(0.7375, 0.8933)
1:5	3	0.8123	0.0331	(0.7344, 0.8902)
pristine	3	0.8386	0.0359	(0.7606, 0.9165)

Pooled StDev = 0.0585153

Tukey Pairwise Comparisons

Grouping Information Using the Tukey Method and 95% Confidence

membrane	N	Mean	Grouping
1:1	3	0.86731	A
pristine	3	0.8386	A
1:10	3	0.8154	A
1:5	3	0.8123	A

Means that do not share a letter are significantly different.

5.3.4 Porosity for 80/20 blend given 1:10 crosslinking and ethanol chemical testing

Method

Null hypothesis	All means are equal
Alternative hypothesis	Not all means are equal
Significance level	α = 0.05

Equal variances were assumed for the analysis.

Factor Information

Factor	Levels	Values
Chemical	2	Ethanol, None

Analysis of Variance

Source	DF	Adj SS	Adj MS	F-Value	P-Value
Chemical	1	0.03976	0.039758	13.69	0.001

Error	22	0.06390	0.002905
Total	23	0.10366	

Model Summary

S	R-sq	R-sq(adj)	R-sq(pred)
0.0538958	38.35%	35.55%	26.64%

Means

Chemical	N	Mean	StDev	95% CI
Ethanol	12	0.7520	0.0528	(0.7197, 0.7843)
None	12	0.8334	0.0550	(0.8011, 0.8657)

Pooled StDev = 0.0538958

Tukey Pairwise Comparisons

Grouping Information Using the Tukey Method and 95% Confidence

Chemical	N	Mean	Grouping	
None	12	0.8334	A	
Ethanol	12	0.7520		B

Means that do not share a letter are significantly different.

5.2.5 Permeance for PCMS given different crosslinking ratios and no chemical testing

Method

Null hypothesis	All means are equal
Alternative hypothesis	Not all means are equal
Significance level	$\alpha = 0.05$

Equal variances were assumed for the analysis.

Factor Information

Factor	Levels	Values
membrane	4	pristine, x1, x10, x5

Analysis of Variance

Source	DF	Adj SS	Adj MS	F-Value	P-Value
membrane	3	965884	321961	7.51	0.010
Error	8	342916	42865		
